# The roots of a colony: establishment and growth of a Magellanic Penguin breeding site in Patagonia, Argentina

**DOI:** 10.7717/peerj.21594

**Published:** 2026-07-23

**Authors:** Candela Tisera Manochio, Laura Marina Reyes, Thomas Mattern, Pablo Garcia Borboroglu

**Affiliations:** 1 Facultad de Ciencias Naturales y Ciencias de la Salud, Universidad Nacional de la Patagonia San Juan Bosco, Puerto Madryn, Chubut, Argentina; 2 Global Penguin Society, Puerto Madryn, Chubut, Argentina; 3 Department of Zoology, University of Otago, Dunedin, Otago, New Zealand; 4 Centro para el Estudio de Sistemas Marinos (CESIMAR), Consejo Nacional de Investigaciones Científicas y Técnicas (CONICET), Puerto Madryn, Chubut, Argentina

**Keywords:** Magellanic penguin, Colony formation, Colony establishment, Abundance, Breeding success, Area occupation, Patagonia

## Abstract

The unique opportunity to record the establishment of seabird colonies provides key insights into population dynamics and habitat use. This is the case of the Magellanic penguin (Spheniscus magellanicus ) colony El Pedral. Located in Patagonia, Argentina, this colony was reported from its first 6 pairs in 2008, and was monitored continuously over 15 years. Annual surveys of breeding pairs were conducted to calculate population growth (*λ*). A remarkable increase in population size, with an average annual growth of 50%, was observed during the study period. Breeding success was measured by recording the number of breeding pairs and fledglings produced, with fluctuations observed between years. Additionally, the expansion of the colony into two main areas was detected, and spatial occupancy was evaluated using GPS mapping. Gradual colonization of available habitat was observed, with the first established sector expanding more rapidly than the other. The rapid growth and expansion, along with the variable breeding success observed in our results, highlight the important contribution of immigration in new colonies.

## Introduction

Colonial breeding in vertebrates is a specialized form of group living in which individuals raise their offspring in densely aggregated territories (Rolland, Danchin & Fraipont, 1998; Wittenberger & Hunt, 1985). This strategy is particularly common among seabirds, 98% of which nest in colonies and regularly depart from them to forage (Wittenberger & Hunt, 1985). Because of their life history traits, seabird metapopulation dynamics are influenced not only by local recruitment but also by the movement of juveniles among colonies (Greenwood, 1980; Inchausti & Weimerskirch, 2002). Colonies may therefore grow through both local recruitment and immigration (Santoro, 2014). In this sense, connectivity between colonies supports the viability of seabird populations, facilitating the establishment of new colonies as well as the expansion of existing ones (Hanski & Gaggioti, 2004).

Despite its importance, the emergence of new colonies has rarely been documented in detail, with only a few studies describing settlement and early growth (Anderson, 1982; Dunlop, 2005; Kildaw et al., 2005; Lisnizer, Garcia-Borboroglu & Yorio, 2011; Martínez-Abraín et al., 2001; Pyk et al., 2013). The recent establishment of a Magellanic penguin (*Spheniscus magellanicus*) colony along the coast of Chubut Province, southern Argentina, provided a unique opportunity to study these processes.

The Magellanic penguin is a seabird widely distributed in southern South America, breeding along the coasts of Argentina and Chile as well as in the Islas Malvinas/Falkland Islands with a global population estimated at ∼1,030,000 pairs (Boersma et al., 2013), a figure likely considerably higher today given recent projections for 2024 of ∼1.28 million breeding pairs in Argentina alone (Hombre, Arias & Carrasco, 2026). In Argentina, 73 colonies are distributed along 4,500 km of Patagonian coastline (Boersma et al., 2013; Garcia Borboroglu et al., 2022; Hombre, Arias & Carrasco, 2026; Raya Rey et al., 2014).

Population trends in Argentina vary regionally. Since the mid-1990s, numbers in the southern and central areas of Chubut Province have declined, with some colonies remaining stable. In contrast, populations in the northern part of the range (northern Chubut and Río Negro) have increased (Boersma et al., 2013; Garcia Borboroglu et al., 2022; Pozzi, 2015). The recent establishment of colonies in Río Negro Province has expanded the species’ distribution northward by approximately one degree of latitude (Garcia Borboroglu et al., 2022).

Within this context of shifting distribution and abundance, the El Pedral colony was founded (Pozzi et al., 2015). Discovered in 2008 and monitored continuously since, it offers an exceptional opportunity to examine the earliest stages of colony development. In this study, we describe the first fifteen years of El Pedral, analyzing (i) changes in abundance and population growth, (ii) breeding success across years, and (iii) spatial expansion and shifts in nesting area. Together, this data provides insight into the demographic and ecological processes that underpin the establishment and growth of a new Magellanic penguin colony.

## Materials & Methods

### Abundance and population trend

This study presents data from 2008 to 2022, including new data collected from 2014 to 2022, and previous data from (Pozzi, 2015; Pozzi et al., 2015). From 2009 to 2022 inclusive, during the late egg incubation stage (late October to early November), the abundance of breeding pairs at El Pedral colony was estimated using direct counts of active nests. A nest was considered active if it contained at least one egg or an adult individual (García Borboroglu et al., 2002; Yorio et al., 1998).

Observers (up to 14) were positioned in a straight line perpendicular to the colony. The distance between each observer was determined *in situ* according to vegetation cover, ensuring visual detection of the nests. Observers advanced at a uniform pace along parallel transects until the entire colony had been surveyed. Each census taker counted all active nests within their assigned established strip using a tally counter.

Survey protocols, sampling design, and survey timing were consistent across both datasets (and subsequent surveys). All counts followed the same standardized methodology (Garcia Borboroglu et al., 2022) and were conducted at comparable stages of the breeding season, ensuring direct comparability of abundance estimates. In small colonies, like El Pedral, direct nest counts are a viable option, as shown in other works (Pozzi et al., 2015; Southwell et al., 2015), and allow a complete census with lower error compared to density estimates.

Based on these abundance data, the instantaneous population growth rate (*r*) was estimated under a linear model fitted with log-transformed abundance, representing an exponential model. The slope of the regression line between the log of the abundance values over time was taken as *r*. Growth between successive time points was described by the finite growth rate (*λ*) calculated as: 

*N*_*t*_ = *N*_0_ × *e* ^*rt*^ with *λ* = *e* ^*r*^

where *N*_*t*_ is the number of breeding pairs in the final year, *N*_0_ is the number in the initial year, *r* is the instantaneous growth rate, and *t*−*t*_0_ is the time interval between estimates (Caughley, 1977). This method assumes deterministic growth, attributing variability to multiplicative observation errors (Hilborn & Mangel, 1997).

**Figure 1:**
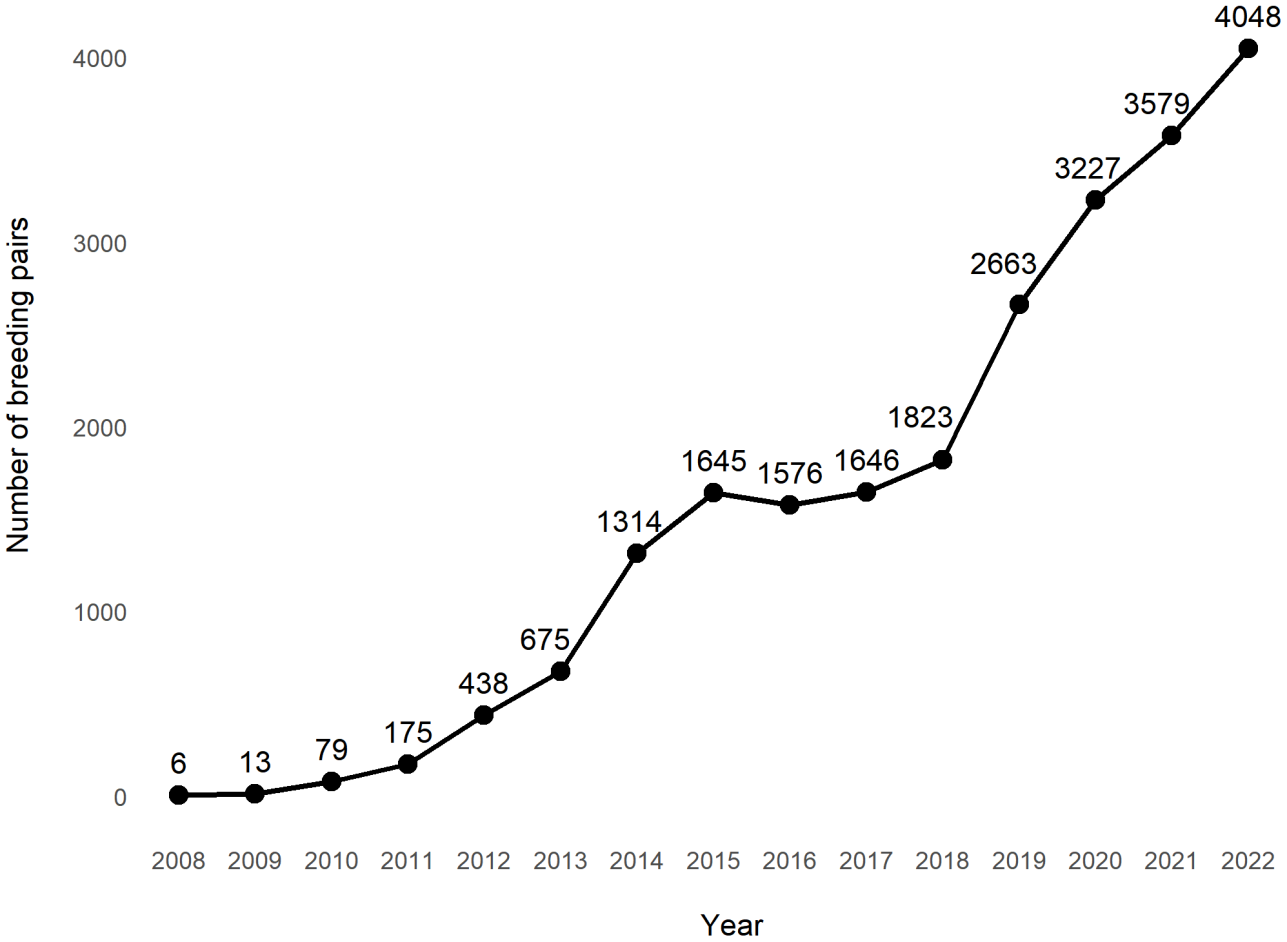
Number of breeding pairs of Magellanic penguins per year in El Pedral colony.

**Figure 2:**
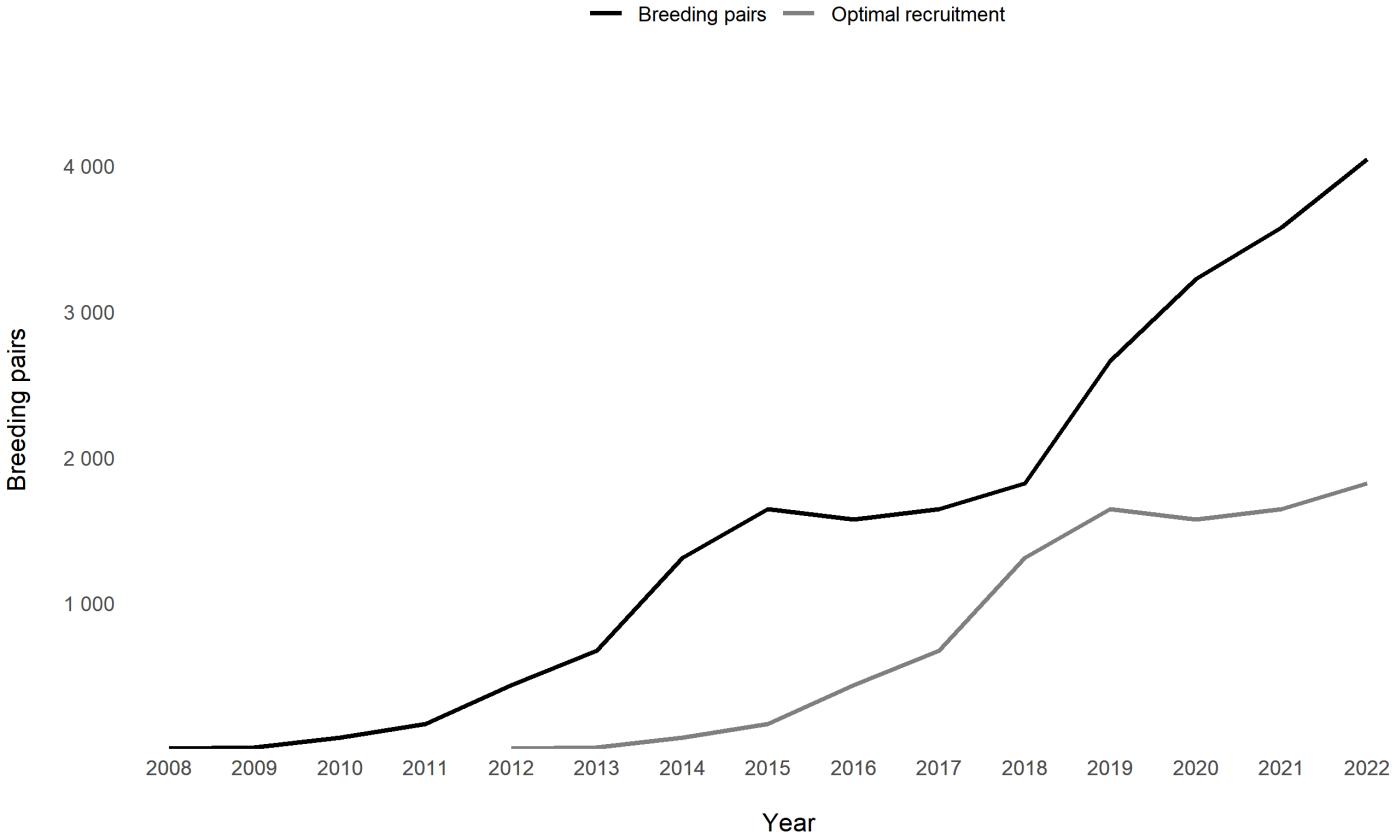
Observed *versus* theoretical increases in colony size. The black line indicates the observed increase, while the grey line represents the theoretical maximum increase from local recruitment, accounting for age at first breeding.

Values of *λ* = 1 indicate a stable population, *λ* < 1 a declining population; and *λ* > 1 an increasing population (Caughley, 1977). Confidence intervals (95%) for growth rates were obtained through bootstrap analysis of regression residuals. All data used for calculations of population growth (*λ*) and Figs. 1 and 2 are included directly in the R script (see File S1), which was implemented in R version 4.4.1 (R Core Team, 2024).

### Breeding success

**Figure 3:**
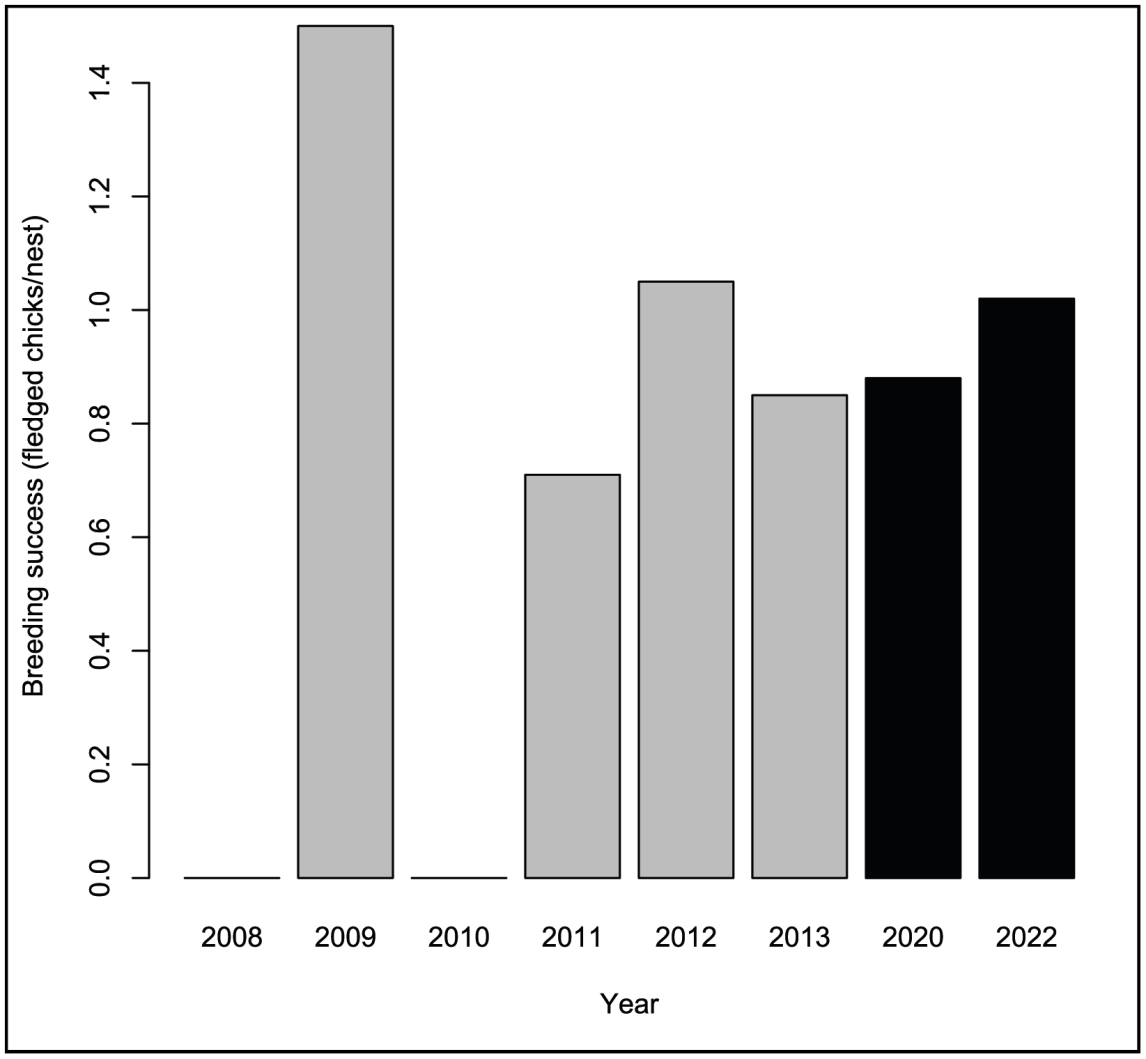
Breeding success of Magellanic penguins per year in El Pedral colony. The years in grey correspond to Pozzi (2015). The years in black correspond to data collected during this study.

Breeding success was calculated as the number of chicks surviving to fledging per nest where at least one egg was laid (Rebstock & Boersma, 2017). It was estimated using two nest visits, following the protocols of Rebstock & Boersma (2017), which align with the breeding phenology of the study area. The first visit occurred between late October and early November, after the peak of laying but before the first chicks hatched. Active nests were randomly chosen, marked with numbered tags, and their contents recorded. The second visit took place in mid-January, when chicks were still in the nest but close to fledging. Additional visits conducted for GPS deployment allowed confirmation that chicks remained associated with their nesting areas at the time of the January survey and had not yet dispersed widely within the colony. A chick was considered fledged if it was observed alive between 10-15 January. The data from 2008 to 2013 come from Pozzi et al. (2015). In 2020 and 2022, field visits were carried out directly by our research team (Table 1). All data used for Fig. 3 is included directly in the R script (see File S1), which was implemented in R version 4.4.1 (R Core Team, 2024).

### Area occupation

**Figure 4:**
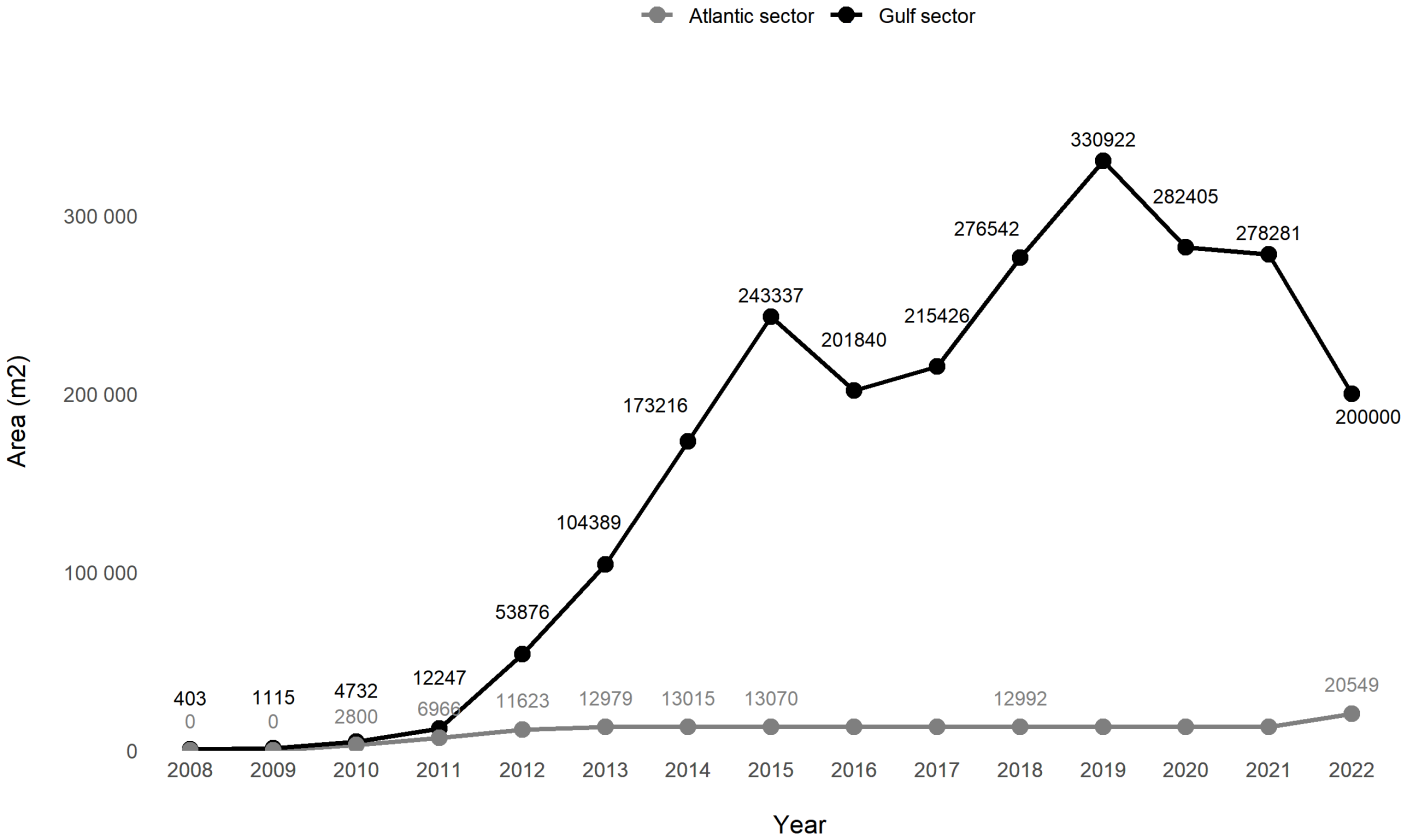
Annual changes in occupied area for each sector of El Pedral colony.

**Figure 5:**
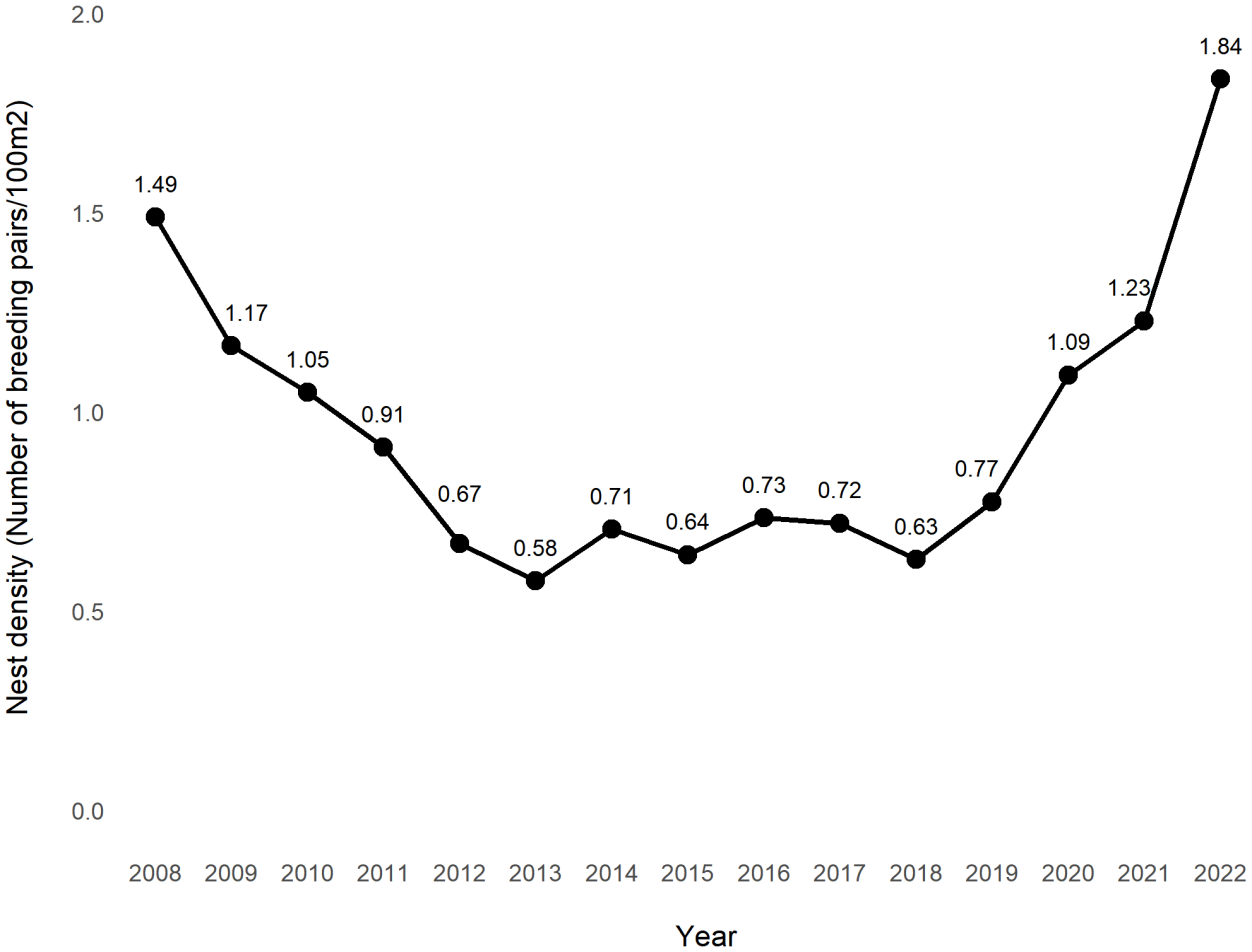
Nesting density expressed by number of nests/100 m^2^.

To map colony extent, the outermost active nests were georeferenced using a handheld GPS device (GPSMAP 64SX, Garmin Ltd, Olathe, Kansas, USA). The colony perimeter was then delineated as polygons in QGIS v3.36.1 (QGIS Development Team, 2024), allowing comparison of occupied areas across years. Geographical features provided natural boundaries for much of the perimeter (cliff to the south, pebble beach to the north, and the shoreline to the east). For 2008, when GPS mapping was not undertaken, the colony area was digitally reconstructed from our records. All data used for Figs. 4 and 5 is included directly in the R script (see File S1), which was implemented in R version 4.4.1 (R Core Team, 2024).

**Table 1:** Key breeding dates and breeding success of Magellanic penguins at El Pedral colony over eight breeding seasons. Data for 2008 were provided by staff at the Estancia and verified by the research team, 2009–2013 from Pozzi et al. (2015); 2020 and 2022 were determined during this study

**Season**	**Nests monitored** *(n)*	**Mean incubation date**	**Mean fledging date**	**Breeding success** *(chicks/nest)*
2008	6	–	–	0
2009	11	30 Oct	15 Jan	1.50
2010	48	29 Oct	10 Jan	0
2011	14	31 Oct	11 Jan	0.71
2012	20	02 Nov	14 Jan	1.05
2013	26	30 Oct	15 Jan	0.85
2020	42	3 Nov	16 Jan	0.88
2022	45	30 Oct	10 Jan	1.02

### Permits and animal ethics

This study complies with relevant national, international, and institutional guidelines regarding animal care. Research activities were conducted under a research permit (Expediente N^∘^ 1607/2022 MAGIyC) issued by the Ministerio de Turismo y Áreas Protegidas and the Dirección de Fauna y Flora Silvestre, which provided institutional oversight and animal welfare approval for the fieldwork.

All procedures complied with current wildlife legislation (Leyes XI N^∘^10 and I N^∘^ 667; Decreto Reglamentario N^∘^ 880/90; Disposiciones N^∘^104-DFyDS and N^∘^47). The study involved exclusively non-invasive field methods. No animals were captured, handled, or manipulated at any stage of the study.

The principles of the 3Rs (Replacement, Reduction, and Refinement) were applied by relying solely on observational methods, minimizing disturbance to breeding birds. Taxon-specific ethical guidelines for the use of wild birds in research were followed throughout the study (Fair et al., 2023).

## Results

### Abundance and population trends

El Pedral colony increased rapidly following its establishment in 2008, rising from 6 breeding pairs to 4,048 pairs by 2022 (Fig. 1). Over the entire study period, the finite population growth rate was *λ* = 1.50 (95% CI [1.36–1.67]) based on a bootstrap resampling of residuals from an exponential growth model fitted to log-transformed abundance data. This corresponds to an average annual increase of 50%. The rate value indicates a remarkable increase in colony size over the study period, although growth was not constant across years. During the early phase (2008–2014) the colony expanded almost exponentially, while after 2015 the rate of increase slowed and the number of breeding pairs approached a more gradual trajectory. Because of the limited sample size, a formal two-phase statistical analysis was not performed here; instead, we address possible explanations for this shift in the Discussion.

### Breeding success

Average breeding success across all monitored seasons (2009–2013, (Pozzi et al., 2015), and 2019-2022 in this study) was 0.73 chicks/nest (Table 1, Fig. 3). In the founding season (2008/2009, 6 pairs), no eggs or chicks were recorded, and in 2010/2011 (66 pairs) complete breeding failure occurred, with no fledged chicks. The highest breeding success was observed in 2009/2010, with 1.5 fledged chicks per pair (*n* = 13). From 2011 onwards, breeding success ranged between 0.71 (2011) to 1.02 chicks per nest (2022).

### Area occupation

**Figure 6:**
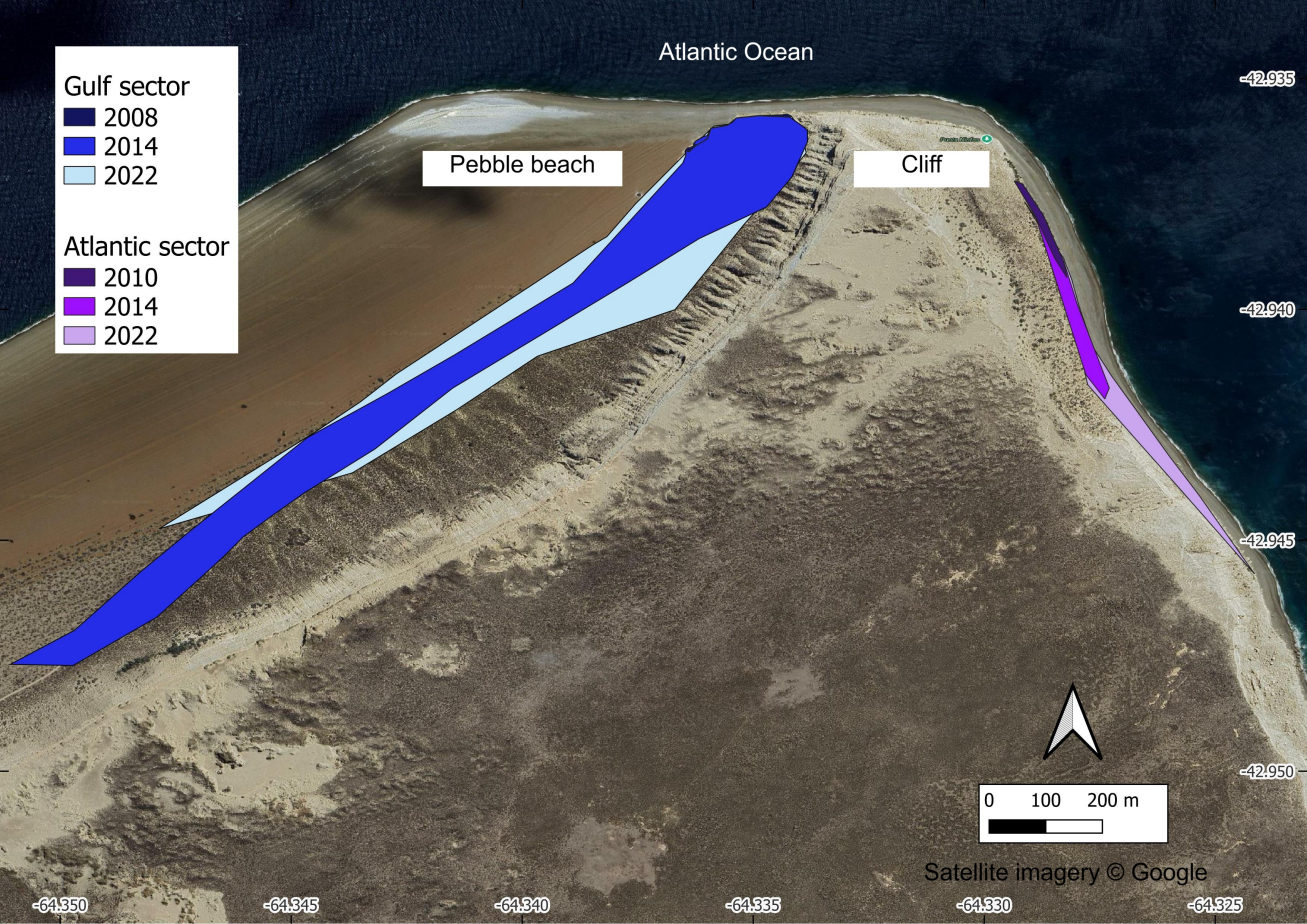
Spatial extent of El Pedral colony over three years. Basemap imagery from Google Satellite (QuickMapServices in QGIS).

From its establishment in 2008 to 2022, the El Pedral colony expanded across two distinct sectors: one bordering Golfo Nuevo (“Gulf Sector”) and another facing the Atlantic Ocean (“Atlantic Sector”). The two areas are separated by a ∼350 m cliff. Colony area was calculated annually for each sector (Figs. 4 and 6; see also animation accessible *via* https://youtu.be/3aS9TzqcvVs).

In 2008, the founding group occupied a 403 m^2^ strip northwest of the present colony, nesting in burrows beneath shrubs. The occupied area expanded rapidly to 53,876 m^2^ by 2012. Between 2013 and 2015, expansion continued southwestward, peaking at 240,000 m^2^ in 2015. The area contracted to 201,000 m^2^ in 2016 and remained relatively stable in 2017.

The Atlantic Sector was first occupied in 2010 and increased steadily until stabilizing at ∼13,000 m^2^ in 2013. By 2022, this sector had expanded further to 20,550 m^2^.

### Nest density

Nesting density was calculated as the ratio between the annual abundance with the corresponding occupied area (Fig. 5). Density rose from 0.57 nests per 100 m^2^ in 2013 to 1.83 nests per 100 m^2^ in 2022 (mean = 0.94 nests per 100 m^2^). Values remained stable at ∼0.68 nests per 100 m^2^ until 2019, when a marked increase occurred, peaking in 2022.

## Discussion

The monitoring of the Magellanic penguin colony at El Pedral revealed a marked increase in population size over the 15 years of study. The average annual growth rate was 50%, with especially rapid expansion during the early years after establishment. Between 2008 and 2015 the colony grew at an annual rate of 153% (*λ* = 2.53). Growth halted in 2015 and a decline was observed in 2016, coinciding with the strong 2015/16 El Niño event, which affected food availability across the Southwest Atlantic (Morley et al., 2025). After this setback the colony resumed expansion, increasing at 15% annually (*λ* = 1.15) from 2015 to 2022. The trajectory suggests that the colony is still in the second stage of colony development (immigration and recruitment), and has not yet reached the density-dependent stabilization phase described for other seabirds (Dunlop, 2005). Continued monitoring will be necessary to determine whether El Pedral eventually follows this pattern or diverges due to species-or site-specific conditions.

Comparable high growth rates have been documented in other recently founded seabird colonies. For example, Caspian gulls (*Larus cachinnans*) increased by 78% annually (Skorka, Wójcik & Martyka, 2005), San Félix terns (*Anous stolidus*) by 41% annually (Dunlop, 2005), and kelp gulls (*Larus dominicanus*) by 31.5% annually during their first seven years (Lisnizer, Garcia-Borboroglu & Yorio, 2011; Lisnizer, García Borboroglu & Yorio, 2014). Among Magellanic penguins, the recently established colony at Complejo Islote Lobos grew at 91% annually over five years (Pozzi et al., 2015). El Pedral, in comparison, showed an exceptional 192% increase during its first 5 years (Pozzi et al., 2015) and 87% over the first decade, underscoring the magnitude of the influx of individuals required to sustain such growth rates.

High early growth in new colonies is generally attributed to immigration rather than local reproduction (Moss, Wanless & Harris, 2002; Nelson, 2002; Oro & Ruxton, 2001). The observed increase therefore cannot be achieved through local recruitment alone; while direct evidence (*e.g.*, mark-recapture data, genetic analysis) is lacking, the demographic constraints strongly suggest that substantial immigration into the colony is the most plausible explanation. Age at first breeding in Magellanic penguins is ≥∼4 years, so offspring from year *t* cannot contribute to breeding pairs in year *t* +1. Even under unrealistically optimistic assumptions (two fledglings per pair, 100% survival to breeding age, 1:1 sex ratio), 13 pairs in 2009 could only produce at most 13 additional pairs - and only several years later (Fig. 2). Contemporary demographic work at Punta Tombo, another Magellanic penguin colony, further shows that juvenile survival is low relative to adults (Gownaris & Boersma, 2019) reinforcing the conclusion that local recruitment contributes minimally to the rapid growth observed at El Pedral. This was also demonstrated by Pozzi et al. (2015) during the colony’s initial years, and the continued rapid growth documented here is consistent with immigration remaining the primary driver. A comparable pattern was reported for a recently established Gentoo penguin colony, where demographic analysis indicated that immigration was necessary to account for the observed population growth (Herman & Lynch, 2022). Immigration reflects connectivity among colonies, with both breeding dispersal (shifts between seasons) and natal dispersal (first-time breeders establishing away from their birthplace) contributing (Greenwood, 1980). For Magellanic penguins, which exhibit strong site fidelity (Boersma, 2008), natal dispersal likely dominates. Large colonies may act as “sources” producing emigrants that settle in smaller “sink” colonies (Bouzat, Walker & Boersma, 2009). Direct evidence of dispersal into the region exists: individuals tagged at Punta Tombo have been recorded breeding at colonies in Península Valdés (Pozzi et al., 2015), demonstrating that inter-colony movement of the kind required to fuel El Pedral’s growth does occur in this population.

Breeding success at El Pedral has been variable, ranging from complete failure (*e.g.*, 2010/2011) to high values of 1.5 chicks per nest (Pozzi et al., 2015). The long-term mean of 0.73 chicks/nest is within the range reported for Argentine colonies: 1.15 at San Lorenzo (Pozzi et al., 2015), 0.56 at Isla Vernaci Norte (Yorio et al., 2001), and 0.03–0.95 at Punta Tombo (Boersma, 2008; Boersma & Stokes, 1995). Low values during the colony’s early years may reflect a large proportion of inexperienced breeders (Kharitonov & Siegel-Causey, 1988), although high success in the second year (Pozzi et al., 2015) complicates this interpretation. Other factors such as predation and heavy rainfall likely contribute. At El Pedral, rainfall-induced cliff avalanches have destroyed nests in recent years, illustrating the role of local hazards (Boersma & Rebstock, 2014).

Patterns of colony expansion did not scale proportionally with abundance. After a rapid expansion of occupied area, the colony contracted in recent years while the number of breeders continued to rise, resulting in higher nest density. Early settlement concentrated in shrub habitats - mainly Jume (*Suaeda divaricata*) and Zampa (*Atriplex lampa*) - along the coastal front of the Gulf sector, followed by occupation of the Atlantic coastal sector. Later expansion reached inland steppe areas, though without clear vegetation or soil differences to explain the sequence. A more detailed study of vegetation and substrate characteristics would help clarify habitat preferences underlying this progression.

The choice of colony location is strongly influenced by proximity to foraging grounds (Kharitonov & Siegel-Causey, 1988). Prior to colony establishment, Argentine anchovy (*Engraulis anchoita*), a key prey species, was abundant near the mouth of Golfo Nuevo, where this colony is found (Buratti, 2008). In its early years, El Pedral penguins undertook short feeding trips, averaging 21.1 ± 5.8 km early in chick rearing and 15.69 ± 6.39 km later (Gómez-Laich, Wilson & Sala, 2015; Pozzi et al., 2015). This contrasts with Punta Tombo, where foraging trips were much longer (61 ± 3.9 km early, 108 ± 47 km late) (Boersma & Rebstock, 2009; Rebstock, Abrahms & Boersma, 2022). Such differences highlight how local prey availability reduces foraging effort, potentially enhancing recruitment and breeding success (Boersma & Rebstock, 2009; Lewis et al., 2006). Ongoing GPS-tracking studies at El Pedral will provide valuable new insights into foraging ecology and its role in sustaining colony growth.

## Conclusions

This study provides the first long-term account of the establishment and growth of the Magellanic penguin colony at El Pedral, documenting both its rapid population increase and the variability in breeding performance during the colony’s first 15 years. In addition to quantifying growth rates, our results highlight how immigration, local breeding success, and habitat use interact during the early stages of colony formation, offering new insights into processes that have rarely been described in detail for seabirds.

Against a backdrop of accelerating environmental change, many seabird species are shifting their breeding distributions in response to altered habitat and prey availability. Understanding the long-term dynamics of newly founded colonies is therefore critical for interpreting how species adapt to changing conditions. The successful establishment and persistence of El Pedral demonstrate both the ecological flexibility of Magellanic penguins and the importance of suitable local conditions—such as prey proximity and nesting habitat—in facilitating colonization.

The continued growth of El Pedral provides a valuable natural experiment for studying the mechanisms of seabird colony formation and resilience. At the same time, its implied dependence on immigration and its exposure to local environmental hazards underscore the vulnerability of new colonies. Long-term monitoring of El Pedral and other emerging colonies will be essential to inform conservation strategies for Magellanic penguins and other seabirds facing rapid environmental change.

## Supplemental Information

10.7717/peerj.21594/supp-1 Supplemental Information 1 R script containing the code used for all the analyses performed for this manuscript, including raw data

## Additional Information and Declarations

### Data Availability

The following information was supplied regarding data availability: The raw data and the script are available in the Supplemental Files.
